# Genetic Ablation of Nrf2 Exacerbates Neuroinflammation in Ocular Autoimmunity

**DOI:** 10.3390/ijms231911715

**Published:** 2022-10-03

**Authors:** Yasuhiko Sato, Shoko Saito, Makiko Nakayama, Sunao Sugita, Akihiko Kudo, Hiroshi Keino

**Affiliations:** 1 Division of Radioisotope Research, Kyorin University School of Medicine, 6-20-2 Shinkawa, Mitaka, Tokyo 181-8611, Japan; 2 Department of Ophthalmology, Kyorin University School of Medicine, 6-20-2 Shinkawa, Mitaka, Tokyo 181-8611, Japan; 3 RIKEN Center for Biosystems Dynamics Research, Laboratory for Retinal Regeneration, 2-2-3 Minatojima-Minamimachi, Kobe 650-0047, Japan; 4 Vision Care Inc., 2-1-8 Minatojima-Minamimachi, Kobe 650-0047, Japan; 5 Department of Microscopic Anatomy, Kyorin University School of Medicine, 6-20-2 Shinkawa, Mitaka, Tokyo 181-8611, Japan

**Keywords:** autoimmune uveitis, Nrf2, neuroinflammation, microglia

## Abstract

Experimental autoimmune uveoretinitis (EAU) is an animal model of non-infectious uveitis and is developed by immunization with retinal antigen, interphotoreceptor retinoid-binding protein (IRBP). Nuclear factor erythroid 2- (NF-E2-) related factor 2 (Nrf2) is responsible for regulating antioxidant and inflammatory responses. In this study, we investigated the role of Nrf2 on the development of EAU. Clinical and pathological examination demonstrated that retinal inflammation was exacerbated in Nrf2 knockout (Nrf2 KO) mice compared to wild type (WT) mice, and the expression of inflammatory cytokines (IFN-γ, IL-6, and IL-17) in the retina was significantly elevated in Nrf2 KO mice. GFAP positive cells (astrocytes) and Iba-1 positive cells (microglia cells) in the retina were more numerous in Nrf2 KO mice compared to WT mice. Furthermore, we examined the suppressive effect of the Nrf2 activator CDDO-Im (2-cyano-3,12 dioxooleana-1,9 dien-28-oyl imidazoline) on the development of EAU. The treatment with CDDO-Im significantly reduced the clinical and pathological score of EAU compared to those of vehicle-treated mice. These findings suggest that Nrf2 plays a regulatory role in the pathogenesis of autoimmune uveoretinitis and the activation of the Nrf2 system may have therapeutic potential for protecting vision from autoimmune neuroinflammation.

## 1. Introduction

Non-infectious uveitis is a leading cause of visual impairment in developed countries [1,2]. Experimental autoimmune uveoretinitis (EAU) is an animal model of non-infectious uveitis and shows many clinical and histological features observed in human uveitis, including sarcoidosis and Behçet’s disease [3]. EAU is developed by immunization with retinal antigen, interphotoreceptor retinoid-binding protein (IRBP), in complete Freund’s adjuvant (CFA), or by adoptive transfer of retinal antigen-specific CD4^+^ T cells [4,5]. EAU is mediated by Th1- a Th17-related immune response against immunized IRBP [6,7], and it is widely used for understanding the pathogenesis of autoimmune uveitis. In addition, EAU is a useful model for the development of new treatment strategies for refractory uveitis [8].

Oxidative stress plays an important role in the pathogenesis of various ocular diseases [9]. A number of studies have demonstrated that reactive oxygen species (ROS) increase in uveitic eyes and that excessive ROS cause tissue damage in uveitis. This suggests that antioxidant therapy could be effective for the suppression of intraocular inflammation and the protection of intraocular tissue damage due to uveitis [10,11,12]. Nuclear factor erythroid 2- (NF-E2-) related factor 2 (Nrf2) is a transcriptional factor that binds to the antioxidant response element (ARE), leading to the activation of cytoprotective genes such as heme oxygenase-1 (HO-1/*Hmox1*) and NAD(P)H: quinone oxidoreductase 1 (NQO1/*Nqo1*) [13,14]. Recent reports have demonstrated that activation of Nrf2 promotes neuroprotective roles and reduces neuroinflammation [14]. In the field of uveitis, a previous study revealed that genetic ablation of Nrf2 exacerbates endotoxin-induced uveitis [15]. However, as far as we know, there are no studies investigating the critical role of Nrf2 on experimental autoimmune uveoretinitis using the genetic knockout mouse model. In the present study, we report that the deficiency of Nrf2 aggravates EAU in mice, and the Nrf2 triterpenoid activator, CDDO-Im (2-cyano-3,12 dioxooleana-1,9 dien-28-oyl imidazoline), ameliorates the severity of EAU.

## 2. Results

### 2.1. Exacerbation of EAU in Nrf2 Deficient Mice

**Figure 1:**
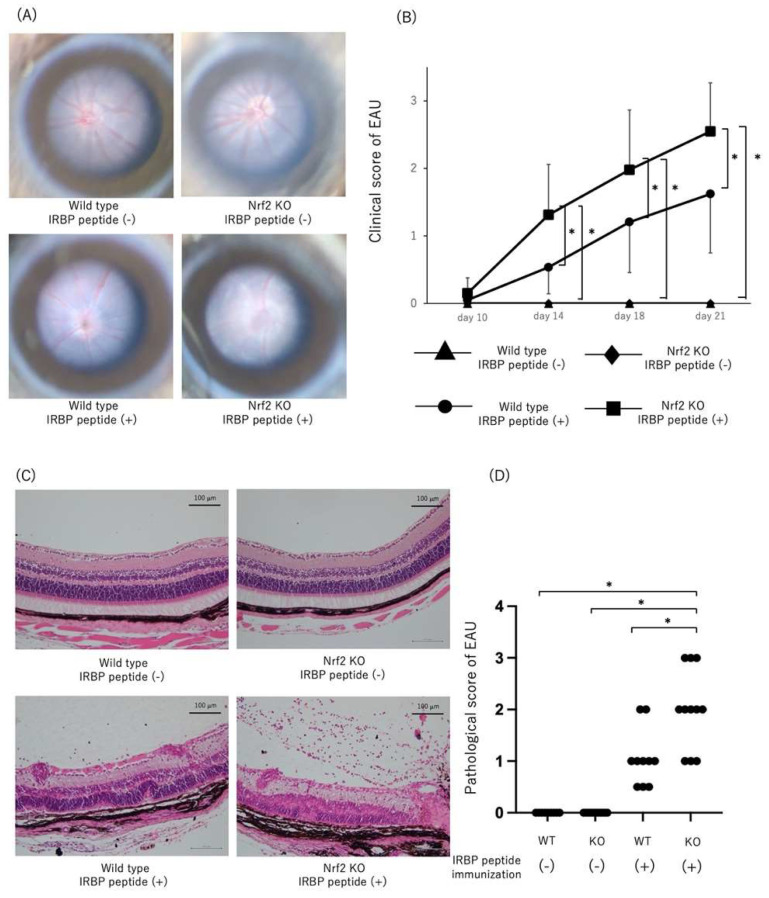
Genetic ablation of Nrf2 exacerbates EAU. Wild type (WT) and Nrf2 knockout (Nrf2 KO) mice were immunized with IRBP peptide, and the clinical score and pathological score were evaluated. (**A**): Fundus images of non-immunized WT mice and Nrf2 KO mice as control and WT mice immunized with IRBP peptide and Nrf2 KO mice immunized with IRBP peptide. Non-immunized WT mice (*n* = 11 mice) and Nrf2 KO mice (*n* = 11 mice) showed no inflammatory changes. WT mice immunized with IRBP peptide (on day 18 after immunization) revealed retinal vasculitis (clinical score: 2) and Nrf2 KO mice immunized with IRBP peptide (on day 18 after immunization) showed retinal vasculitis and retinal exudates (clinical score: 3). (**B**): The changes of clinical score of EAU in non-immunized WT mice (*n* = 11 mice), non-immunized Nrf2 KO mice (*n* = 11 mice), immunized WT mice (*n* = 22 mice), and immunized Nrf2 KO mice (*n* = 22 mice). Clinical score was evaluated on days 10, 14, 18, and 21 after immunization. The mean ± standard deviation for each group is shown. Asterisk indicates *p* < 0.05 versus Nrf2 KO mice immunized with IRBP peptide. Statistical analysis was performed according to ANOVA followed by Bonferroni test. (**C**): HE sections of non-immunized WT mice and Nrf2 KO mice and WT mice immunized with IRBP peptide and Nrf2 KO mice immunized with IRBP peptide. Non-immunized WT mice (*n* = 9 mice) and Nrf2 KO mice (*n* = 11 mice) showed no inflammatory cells in vitreous and retina. Nrf2 KO mice immunized with IRBP peptide revealed enhanced inflammation in the posterior segment with severe vitreous infiltration and destruction of outer retinal layer compared to those of WT mice immunized with IRBP peptide. (**D**): Histopathological score of EAU in non-immunized WT mice (*n* = 9 mice), non-immunized Nrf2 KO mice (*n* = 11 mice), immunized WT mice (*n* = 10 mice), and immunized Nrf2 KO mice (*n* = 11 mice) on day 21. Either eye per mouse was used for histopathological analysis. The mean ± standard deviation for each group is shown. Asterisk indicates *p* < 0.05 versus Nrf2 KO mice immunized with IRBP peptide. Statistical analysis was performed according to ANOVA followed by Bonferroni test. Scale bar: 100 μm.

**Figure 2:**
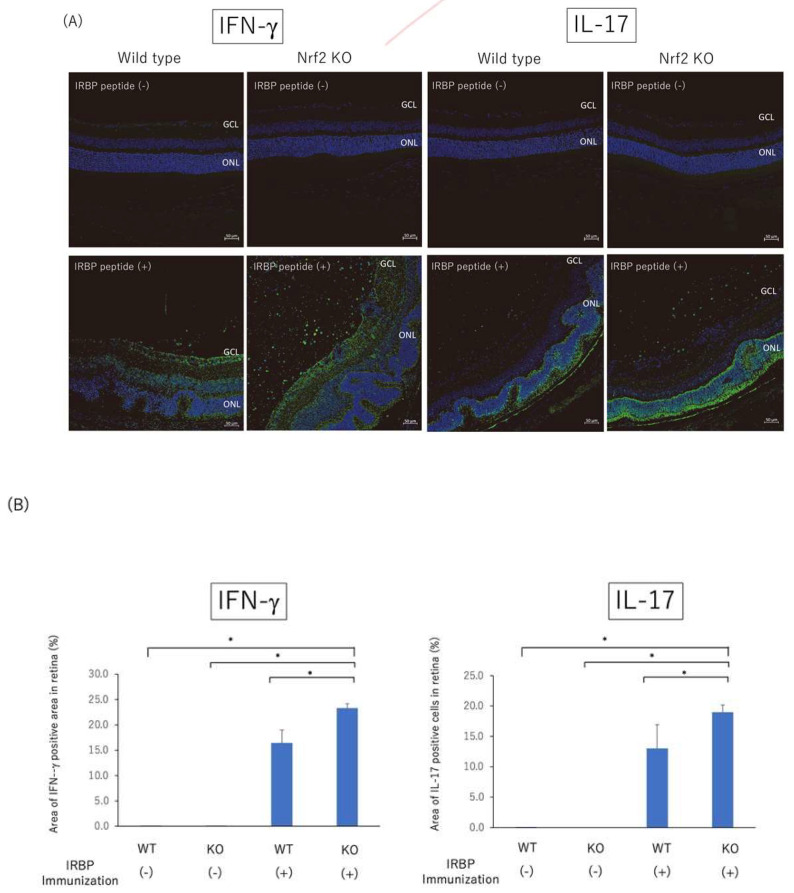
Immunohistochemical analysis of IFN-γ and IL-17 positive cells (green) in posterior segment of non-immunized WT mice, non-immunized Nrf2 KO mice, immunized WT mice, and immunized Nrf2 KO mice on day 21 after immunization. Nuclei were labeled with DAPI (blue). Wild type and Nrf2 knockout mice were immunized with IRBP peptide and immunohistochemical analysis was performed using eyes enucleated on day 21 after immunization. (**A**) Photomicrographs of the posterior segment show IFN-γ and IL-17 positive cells in non-immunized WT mice, non-immunized Nrf2 KO mice, immunized WT mice, and immunized Nrf2 KO mice. Scale bar: 50 μm. GCL: ganglion cell layer, ONL: outer nuclear layer. (**B**) The relative area (%) of IFN-γ positive cells or IL-17 positive cells in a given area of retina was measured using binary images. The mean ± standard deviation for each group (*n* = 3–4 mice per group) is shown. Asterisk indicates *p* < 0.05 versus Nrf2 KO mice immunized with IRBP peptide. Statistical analysis was performed according to ANOVA followed by Bonferroni test.

To determine the effect on the development of EAU by genetic ablation of Nrf2, both wild type (WT) and Nrf2 knockout (Nrf2 KO) mice were immunized with IRBP peptide to evaluate the absence of Nrf2 on the clinical and pathological score of EAU. As shown in Figure 1A, fundus images of non-immunized WT mice and Nrf2KO mice as controls demonstrated no inflammatory changes. The fundus images of Nrf2KO mice immunized with IRBP peptide revealed severe retinal vasculitis and multiple exudates compared to WT mice immunized with IRBP. As shown in Figure 1B, the clinical score of EAU was significantly higher in Nrf2 KO mice compared to non-immunized WT mice, non-immunized Nrf2 KO mice, and immunized WT mice on days 14, 18, and 21 after immunization. As shown in Figure 1C,D, the histopathological analysis showed no inflammatory cells in the vitreous and retina in non-immunized WT mice and Nrf2KO mice. The histopathological score of EAU was significantly higher in Nrf2 KO mice immunized with IRBP peptide compared to those of non-immunized WT mice, non-immunized Nrf2 KO mice, and immunized WT mice. The hematoxylin/eosin (HE) sections in Nrf2 KO mice on day 21 after immunization revealed enhanced inflammation in the posterior segment with severe vitreous infiltration and destruction of the outer retinal layer. Furthermore, as shown in Figure 2A,B, immunohistochemical analysis revealed that the relative area of IFN-γ positive cells in a given area of the retina was significantly greater in the retinas of Nrf2 KO mice immunized with IRBP peptide compared to those of non-immunized WT mice, non-immunized Nrf2 KO mice, and immunized WT mice. In addition, the relative area of IFN-γ positive cells in the inner layer (ganglion cell layer~inner plexiform layer) was significantly greater in immunized Nrf2 KO mice compared to those of immunized WT mice, whereas there was no significant difference in both mid-retina and outer retina between immunized WT mice and immunized Nrf2 KO mice. The relative area of IL-17 positive cells in a given area of the retina was also significantly greater in the retinas of immunized Nrf2 KO mice compared to those of non-immunized WT mice, non-immunized Nrf2 KO mice, and immunized WT mice. The relative area of IL-17 positive cells in both mid-retina and outer retina was significantly greater in immunized Nrf2 KO mice, whereas there was no significant difference in the inner layer between immunized WT mice and immunized Nrf2 KO mice.

**Figure 3:**
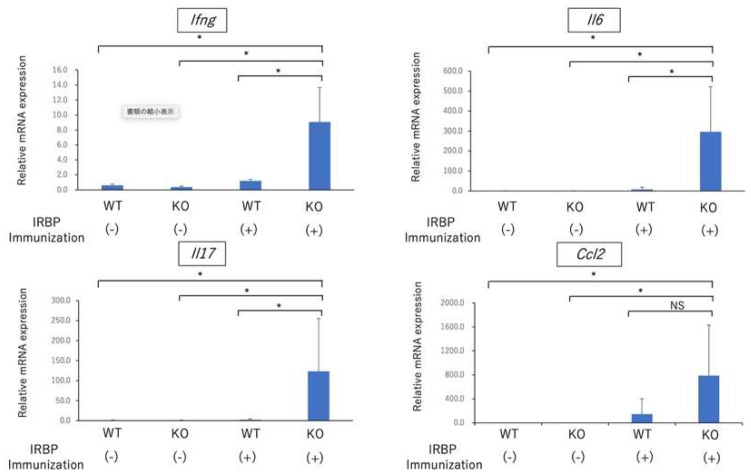
The expression of inflammatory cytokines in retina at early phase of EAU. Wild type (WT) and Nrf2 knockout (Nrf2 KO) mice were immunized with IRBP peptide, and retina was collected from enucleated eyes on day 14 after immunization. Total RNA was extracted from retina of non-immunized WT mice, non-immunized Nrf2KO mice, WT mice immunized with IRBP peptide and Nrf2KO mice immunized with IRBP peptide. The quantitative PCR was performed regarding the gene expression of *Ifng, Il17, Il6, and Ccl2* (MCP-1). The mean ± standard deviation for each group (*n* = 4–5 mice per group) is shown. Asterisk indicates *p* < 0.05 versus WT mice. Statistical analysis was performed according to ANOVA followed by Bonferroni test. NS: not significant.

Recent reports have demonstrated that Nrf2 elicits an anti-inflammatory response by repressing the expression of several pro-inflammatory cytokines in macrophages, monocytes, microglia, and astrocytes [14,16]. Next, we compared the expression of *Ifng, Il6, Il17,* and chemokine (C-C motif) ligand 2 gene/Monocyte chemoattractant protein-1 gene (*Ccl2*/MCP-1) in the retina at the early phase of EAU (on day 14) between WT mice and Nrf2 mice. As shown in Figure 3, the expression of *Ifng, Il6, and Il17* was significantly higher in the retina of Nrf2 KO mice immunized with IRBP peptide compared to those of WT mice immunized with IRBP peptide, non-immunized WT mice, and Nrf2 KO mice. These results demonstrated that proinflammatory cytokines including *Ifng, Il6, and Il17* were significantly elevated in the retina from Nrf2 KO immunized with IRBP peptide, indicating that Nrf2 may play an important role in the regulation of the expression of proinflammatory cytokines in the retina at the early phase of EAU.

**Figure 4:**
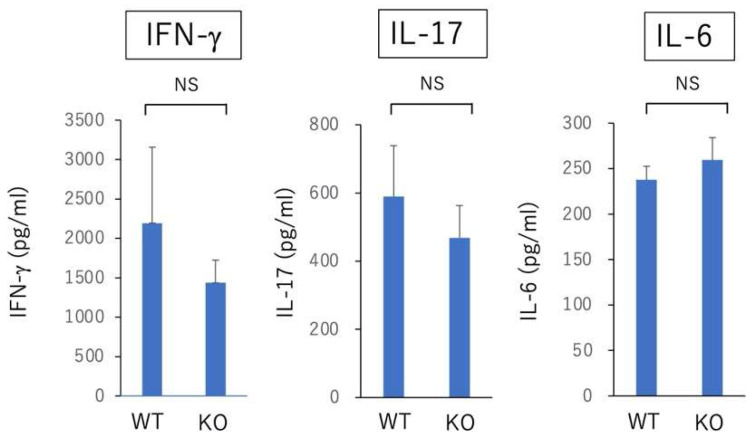
In vitro analysis of Th1 and Th17 immune responses of draining lymph node cells in EAU-induced WT mice and Nrf2 KO mice. Wild type (WT) and Nrf2 knockout (Nrf2 KO) mice were immunized with IRBP peptide and draining lymph node cells were collected on day 14 after immunization and pooled within groups. Each group consisted of 4 mice. The draining lymph node cells were cultured with IRBP peptide for 72 h. The level of IFN-γ, IL-6, and Il-17 in supernatant of cultured wells was measured using ELISA method. The mean ± standard deviation for each group (*n* = 4 wells per group) is shown. Statistical analysis was performed according to Student’s *t* test. NS: not significant.

As described earlier, rodent EAU is mediated by Th1 and Th17 immune response against immunized retinal antigen [6,7]. We determined the effect of deficiency of Nrf2 on the systemic Th1 and Th17 immune responses in EAU-induced mice. Draining lymph node cells on day 14 after immunization were collected from both WT mice and Nrf2 KO mice and cultured with IRBP peptide for 72 h. The level of IFN-γ, IL-6, and Il-17 in the supernatant of cultured wells was measured using the ELISA method. As shown in Figure 4, there was no significant difference between WT mice and Nrf2 KO mice with regard to the expression of all cytokines. 

### 2.2. GFAP and Iba-1 Expression in Retina of EAU-Induced Nrf2 Deficient Mice

**Figure 5:**
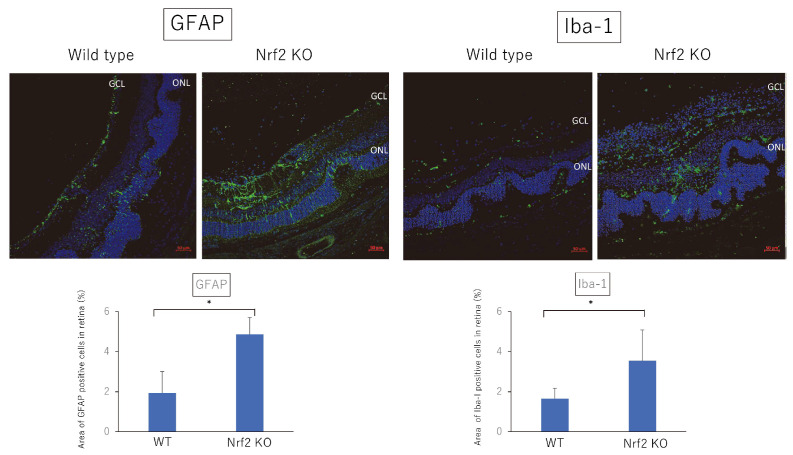
Immunohistochemical analysis of GFAP and Iba-1 positive cells in posterior segment of EAU-induced WT mice and Nrf2 KO mice. Wild type and Nrf2 knockout mice were immunized with IRBP peptide and immunohistochemical analysis was performed using eyes enucleated on day 21 after immunization. Green: GFAP and Iba-1 positive cells. Blue: Nuclei. Scale bar: 50 μm. GCL: ganglion cell layer, ONL: outer nuclear layer. Either eye per mouse was used for immunohistochemical analysis. The mean ± standard deviation for each group (*n* = 3–6 mice per group) is shown. The relative area (%) of GFAP positive cells or Iba-1 positive cells in a given area of retina was calculated using binary images. Asterisk indicates *p* < 0.05 versus WT mice. Statistical analysis was performed according to Student’s *t* test.

We next examined whether the enhanced retinal inflammation in Nrf2 KO mice with EAU was associated with activated astrocytes and microglia. We performed the immunohistochemical analysis to investigate the localization of microglia and astrocytes using Iba-1+ (microglia marker) and glial fibrillary acidic protein (GFAP) (astrocyte marker) in the posterior segment of EAU-induced WT mice and Nrf2 KO mice. As shown in Figure 5, GFAP-positive cells were observed in the inner layer of the retinas of WT mice, whereas GFAP-positive cells were more numerous in Nrf2 KO mice. Furthermore, more Iba-1 positive cells are observed in the retinas of Nrf2 KO mice compared to that of WT mice. The relative area (%) of GFAP-positive cells and Iba-1-positive cells was significantly greater in the retinas of Nrf2 KO mice. These findings suggest that the deficiency of Nrf2 promotes gliosis in retinal tissue and enhances the activation of microglia in EAU-induced mice. 

### 2.3. Amelioration of EAU by CDDO-Im Treatment 

**Figure 6:**
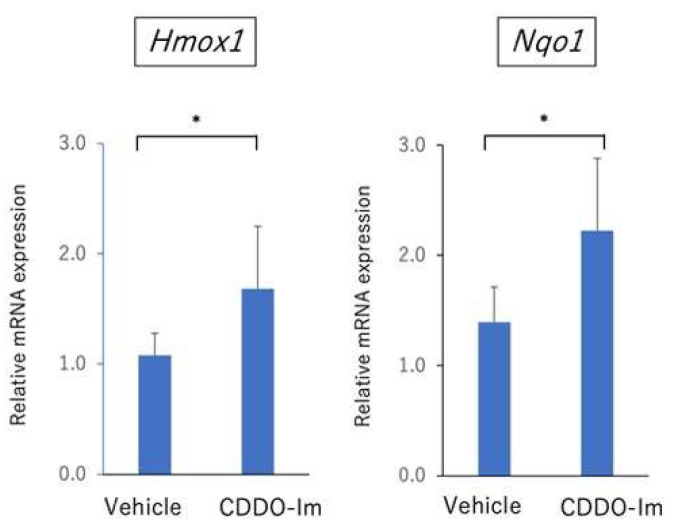
The expression of antioxidants (*Hmox1* and *Nqo1*) in retina of CDDO-Im-treated mice. Mice received vehicle or CDDO-Im on days 0, 2, 4, 6, 8, 10, 12, and 14, and eyes were enucleated on day 14. The expression of (*Hmox1* and *Nqo1*) in retina was examined by quantitative PCR. The mean ± standard deviation for each group (*n* = 6 mice per group) is shown. Asterisk indicates *p* < 0.05 versus vehicle-treated mice. Statistical analysis was performed according to Student’s *t* test.

CDDO-Im is known to be a Nrf2 triterpenoid activator and leads to the activation of Nrf2/HO-1 signaling [17,18,19]. First, to examine the effect of antioxidant activity of CDDO-Im, mice received vehicle or CDDO-Im on days 0, 2, 4, 6, 8, 10, 12, and 14, and the eyes were enucleated on day 14. The expression of *Hmox1* and *Nqo1* in the retina was examined by quantitative PCR. As shown in Figure 6, treatment of CDDO-Im significantly increased the expression of *Hmox1* and *Nqo1* in the retina. These results indicate that CDDO-Im has the capacity to promote the expression of antioxidant genes in the retina.

**Figure 7:**
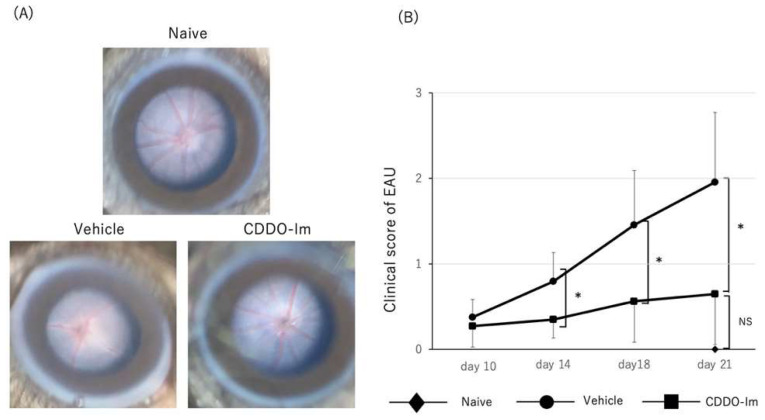

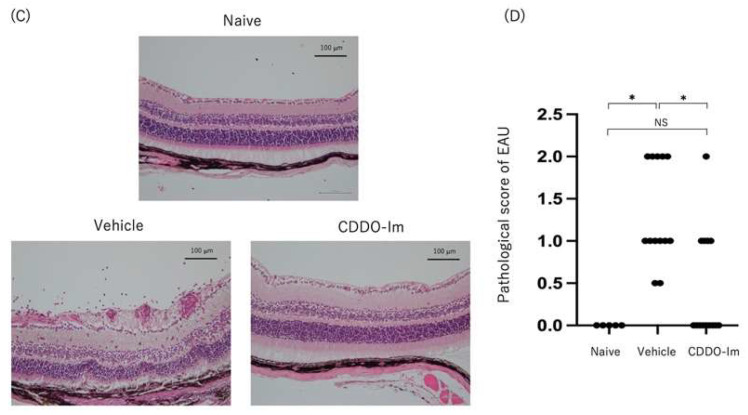
Amelioration of EAU by CDDO-Im treatment. Mice were immunized with IRBP peptide and vehicle or CDDO-Im was administered on days 0, 2, 4, 6, 8, 10, 12, and 14 after immunization. (**A**): Fundus images and clinical score of EAU in naïve mice (*n* = 5), vehicle-treated mice (*n* = 34 mice) and CDDO-Im-treated mice (*n* = 33 mice). Vehicle-treated mice (on day 18 after immunization) revealed severe retinal vasculitis (clinical score: 3) and CDDO-Im-treated mice (on day 18 after immunization) showed mild retinal vasculitis (clinical score: 1). Naïve mice showed no inflammatory changes on day 21. (**B**): Clinical score was evaluated on days 10, 14, 18, and 21 after immunization. The mean ± standard deviation for each group is shown. Asterisk indicates *p* < 0.05 versus vehicle-treated mice. Statistical analysis was performed according to ANOVA followed by Bonferroni tests. (**C**,**D**): HE sections and histopathological score of EAU in naïve mice (*n* = 5 mice), vehicle-treated mice (*n* = 13 mice) and CDDO-Im-treated mice (*n* = 14 mice) on day 21. Either eye per mouse was used for histopathological analysis. The mean ± standard deviation for each group is shown. Asterisk indicates *p* < 0.05 versus vehicle-treated mice. Statistical analysis was performed according to ANOVA followed by Bonferroni test. Scale bar: 100 μm. NS: not significant.

Next, we determined the suppressive effect of CDDO-Im on the development of EAU and cytokine expression in the retina. We administered CDDO-Im or vehicle on days 0, 2, 4, 6, 8, 10, 12, and 14 after immunization. As shown in Figure 7A,B, fundus images revealed that the clinical score of EAU was significantly lower in CDDO-Im-treated mice compared to those of vehicle-treated mice on days 14, 18, and 21. There was no significant difference in the clinical scores of EAU between naïve mice (non-immunized mice) and CDDO-Im-treated mice on day 21.

Consistent with the results of the clinical score, as shown in Figure 7C,D, the histopathological score of EAU was significantly lower in CDDO-Im-treated mice compared to vehicle-treated mice. The HE sections in vehicle-treated mice on day 21 revealed retinal vasculitis and retinal folds in the posterior segment, whereas CDDO-Im-treated mice showed a relatively normal retinal structure without obvious inflammatory changes. Naïve mice (non-immunized mice) showed no inflammatory cells in the vitreous and retina. These findings suggest that treatment with CDDO-Im ameliorated the severity of EAU in mice.

**Figure 8:**
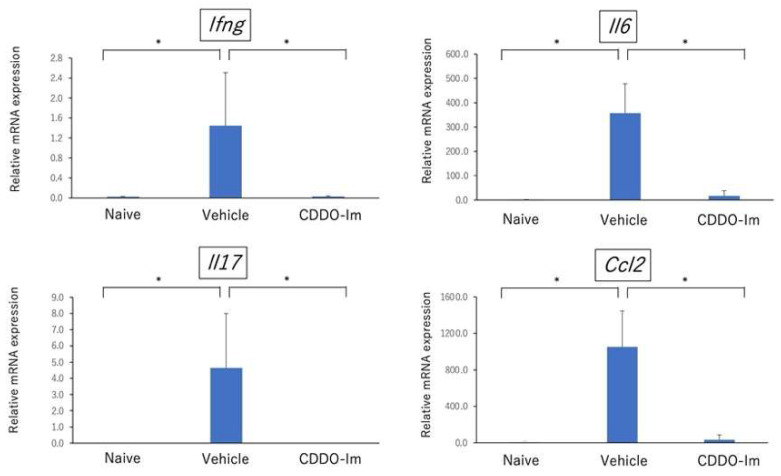
Quantitative PCR analysis of the gene expression of cytokines including *Ifng, Il6, Il17, and Ccl2* in retina in naïve mice (non-immunization), vehicle-treated mice and CDDO-Im-treated mice. Mice were immunized with IRBP peptide and vehicle or CDDO-Im was administered on days 0, 2, 4, 6, 8, 10, 12, and 14 after immunization, and eyes were enucleated on day 21. The expression of *Ifng, Il6, Il17,* and *Ccl2* in retina was examined by quantitative PCR. The mean ± standard deviation for each group (*n* = 5 mice per group) is shown. Asterisk indicates *p* < 0.05 versus vehicle-treated mice. Statistical analysis was performed according to ANOVA followed by Bonferroni tests.

Furthermore, we performed quantitative PCR analysis to compare the gene expression of cytokines including *Ifng*, *Il6*, *Il17*, and *Ccl2* in the retina after immunization between CDDO-Im-treated mice and vehicle-treated mice. As shown in Figure 8, the gene expression of all cytokines was significantly reduced in CDDO-Im-treated mice. These findings are compatible with pathological examination showing that the number of infiltrating inflammatory cells in intraocular tissue was lower in CDDO-Im-treated mice. These results suggest that retinal inflammatory responses were regulated by treatment with CDDO-Im.

**Figure 9:**
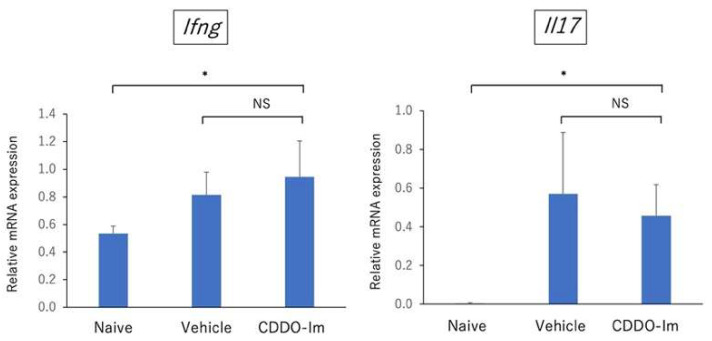
Quantitative PCR analysis of the gene expression of cytokines including *Ifng* and *Il17* in draining lymph node cells in naïve mice (non-immunization), vehicle-treated mice and CDDO-Im-treated mice. Mice were immunized with IRBP peptide and vehicle or CDDO-Im was administered on days 0, 2, 4, 6, 8, 10, 12, and 14 after immunization, and draining lymph node cells were collected on day 21. The gene expression of *Ifng* and *Il17* in draining lymph node cells was examined by quantitative PCR. The mean ± standard deviation for each group (*n* = 5 mice per group) are shown. Asterisk indicates *p* < 0.05 versus CDDO-Im-treated mice. Statistical analysis was performed according to ANOVA followed by Bonferroni tests. NS: not significant.

In addition, to investigate the systemic effect of CDDO-Im on EAU-induced mice, we compared the gene expression of *Ifng* (Th1 signature cytokine) and *Il17* (Th17 signature cytokine) in draining lymph node cells using quantitative PCR between naïve mice, vehicle-treated mice, and CDDO-Im-treated mice on day 21 after immunization. As shown in Figure 9, the qPCR analysis revealed that there was no significant difference regarding the expression of *Ifng* and *Il17* in draining lymph node cells between vehicle-treated mice and CDDO-Im-treated mice, suggesting that systemic administration of CDDO-Im may have no significant effect on the Th1 and Th17 immune responses on EAU-induced mice.

## 3. Discussion

In the present study, we investigated the effect of Nrf2 on the development of EAU. Our results demonstrated that retinal inflammation was exacerbated in Nrf2 KO mice and the expression of inflammatory cytokines in the retina was significantly elevated in Nrf2 KO mice. In addition, GFAP positive cells (astrocytes) and Iba-1 positive cells (microglia cells) were more numerous in Nrf2 KO mice compared to wild type mice. Furthermore, we examined the suppressive effect of the Nrf2 activator, CDDO-Im, on the development of EAU. Treatment with CDDO-Im significantly reduced the clinical and pathological score of EAU compared to those of vehicle-treated mice. These findings suggest that Nrf2 plays a critical role in the pathogenesis of autoimmune uveoretinitis and that Nrf2 may be a potential target molecule for the treatment of refractory intraocular inflammation.

Although there is accumulating evidence showing that the Nrf2/ARE system regulates oxidative stress and has neuroprotective effects on the central nervous system (CNS), recent studies have demonstrated that the Nrf2/ARE system exerts anti-inflammatory responses by repressing the expression of inflammatory cytokines such as IL-6 and IL-1β [16,20] and negatively regulates nuclear factor kappa B (NF-κB) mediated pro-inflammatory cytokine genes, including MCP-1 [20]. In line with the findings above, the present study indicated that retinal inflammation in EAU-induced mice was aggravated in Nrf2 deficient mice and that the mRNA expression level of proinflammatory cytokines, including *Il6*, as well as Th1 related cytokine (*Ifng*) and Th17 related cytokine (*Il17*), in the retina were significantly higher in Nrf2 deficient mice. In addition, the Nrf2 activator, synthetic triterpenoid CDDO-Im, suppressed neuroinflammation and reduced the expression of *Il6*, *Ccl2, Ifng*, and *Il17*. These findings demonstrate that Nrf2 plays a crucial role in the regulation of neuroinflammation in ocular autoimmunity.

Microglia are categorized into two main polarization phenotypes: M1-polarized microglia and M2-polarized microglia [21]. M1-polarized microglia play proinflammatory roles and produce proinflammatory cytokines, including IL-1, IL-6, TNF-α, MCP-1 as well as ROS, whereas M2 is involved in the anti-inflammatory/immunoregulatory process and expresses anti-inflammatory cytokines, such as TGF-β, IL4, and IL-10 [21,22]. In the present study, microglia cells represented by Iba-1 positive cells and astrocytes represented by GFAP positive cells markedly increased in Nrf2 KO mice compared to WT mice. These findings are compatible with previous studies using a mouse model of Alzheimer’s disease showing that Nrf2 deficiency correlated with exacerbated microgliosis and astrogliosis, as determined by an elevation of gene expression of Iba-1 and GFAP levels [23], and an increase in the number of both Iba-1 positive cells and GFAP positive cells [24]. Furthermore, the present study revealed that *Il6* in retina were significantly higher in Nrf2-deficient mice at the early phase of EAU, whereas the levels of *Il6* and *Ccl2* in the retina were markedly inhibited in CDDO-Im-treated mice. These results suggest that deficiency of Nrf2 KO may promote M1 microglia polarization and Nrf2 activation by CDDO-Im may lead to the suppression of M1 polarization. A recent study demonstrated that retinal microglia cells play a critical role in regulating the infiltration of inflammatory cells into the retina of EAU [25]. Thus, targeting the regulation of microglia polarization by Nrf2 may be an important therapeutic strategy for autoimmune neuroinflammation.

Nrf2 regulates the expression of several molecules involved in the cellular defense against oxidative stress and inflammation, including HO-1 and NQO-1 [26]. Both HO-1 and NQO-1 play a role as antioxidants and have anti-inflammatory properties [20]. Our results revealed that treatment with CDDO-Im significantly elevated the expression of *Hmox1* and *Nqo1* in the retina. This finding is in agreement with previous studies showing that CDDO-Im induces HO-1 via the Nrf2/ARE system [15,27]. Jang and colleagues showed that an inducer of HO-1 ameliorated the clinical signs of EAU, whereas an HO-1 inhibitor exacerbated EAU [28]. A previous study revealed that the levels of HO-1 in peripheral blood were significantly lower in patients with active Behçet’s disease compared to healthy group and patients with inactive Behçet’s disease [29]. Further study is required to confirm the antioxidant and anti-inflammatory effects of HO-1 on autoimmune uveitis.

In the present study, to address whether the absence of Nrf2 has an effect on the development of antigen-specific Th1 and Th17 immune response, we compared the level of IFN-γ, IL-17, and IL-6 in the supernatant from cultured lymph node cells stimulated by IRBP peptide between wild type mice and Nrf2 KO mice. The result revealed that there was no significant difference regarding the three cytokines between WT and Nrf2 KO mice, suggesting that the absence of Nrf2 may not have an effect on the development of systemic antigen-specific immune responses. Larabee and colleagues demonstrated that the loss of Nrf2 exacerbated the optic neuritis elicited by experimental autoimmune encephalomyelitis (EAE) [30]. Moreover, they compared the in-vitro cytokine production of IL-6, IL-17, and IFN-γ in cultured spleen cells stimulated with myelin oligodendrocyte glycoprotein (MOG) and showed that the IFN-γ production was significantly elevated in Nrf2 KO mice, whereas there was no significant difference regarding the production of IL-6 and IL-17 between Nrf2KO and wild type mice. Further study is required to determine whether the loss of Nrf2 expression has an association with the development of antigen-specific immune responses.

The quantitative analysis using immunohistochemical sections revealed that the relative area of IFN-γ positive cells in the retina was significantly greater in Nrf2 KO mice compared to those of wild type mice. In addition, the relative area of IFN-γ positive cells in the inner retinal layer (ganglion cell layer~inner plexiform layer) was significantly greater in Nrf2 KO mice, whereas there was no significant difference in both mid-retina and outer retina between WT mice and Nrf2 KO mice. These findings may be due to the accumulation of infiltrating IFN-γ positive macrophages (M1 type macrophages) into inner-retina from retinal vessels or activated M1 microglia in the retina of Nrf2 KO mice may contribute to the production of IFN-γ in the retina.

In summary, our results demonstrated that deficiency of Nrf2 aggravated the neuroinflammation in ocular autoimmunity and that the Nrf2 activator, CDDO-Im, ameliorated neuroinflammation and intraocular tissue damage. These findings support the notion that the activation of the Nrf2/ARE system may have therapeutic potential for protecting vision from autoimmune neuroinflammation.

## 4. Materials and Methods

### 4.1. Mice

This study was approved by Kyorin University Animal Care and Use Committee (protocol number:160). All mice were treated in accordance with institutional guidelines regarding animal experimentation. We obtained C57BL/6J mice from Oriental Yeast Co., Ltd. (Tokyo, Japan). Nrf2−/− (KO) mice were obtained from the Jackson Laboratory (J017009, Jackson Laboratory, Bar Harbor, ME, USA) and housed under specific pathogen-free conditions. Nrf2 KO were backcrossed onto the C57/BL6J mice for five generations. They were genotyped by PCR analysis of tail genomic DNA.

### 4.2. Induction and Scoring of EAU

For induction of EAU, C57BL/6J (wild type: WT, 6–10 weeks) and Nrf2KO mice (6–10 weeks) on C57BL/6J background were immunized subcutaneously in the neck region with 200 µg of IRBP_1-20_ emulsified in 0.2 mL of complete Freund’s adjuvant (Difco, Detroit, MI, USA) containing 1 mg of *Mycobacterium tuberculosis* strain H37Ra (Difco) and 1 μg of pertussis toxin (Sigma Aldrich, St. Louis, MO, USA) in 0.2 mL of PBS was also injected intraperitoneally as an additional adjuvant [31]. IRBP peptide _1-20_ (GPTHLFQPSLVLDMAKVLLD) was obtained from Eurofins Genomics (Tokyo, Japan). Funduscopic examinations were performed on days 10, 14, 18, and 21 after immunization, and the clinical scoring was graded as described in detail [32]. The maximum clinical score from either eye per mouse was recorded for each day of observation. Either eye per mouse was enucleated on day 21 and fixed in 10% neutral buffered formalin and sections were embedded in paraffin and stained with hematoxylin and eosin. The severity of EAU in the posterior segment was assessed histopathologically in a semiquantitative system [3]. CDDO-Im was purchased from Selleck.co.jp (Tokyo, Japan) and DMSO was obtained from Sigma (St Louis, MO, USA). CDDO-Im was solved in DMSO (vehicle) and stored in aliquots at −30 °C before use. Mice received CDDO-Im by gastric gavage (25 μmol/kg body weight) in 200 μl containing 5% DMSO and 95% corn oil (FUJIFILM Wako chemicals, Tokyo, Japan) as vehicle. Control mice received the same volume of vehicle alone. The dose of CDDO-Im was defined based on previous studies showing the anti-inflammatory effect of CDDO-Im [17,33]. The mice received CDDO-Im or vehicle on days 0, 2, 4, 6, 8, 10, 12, and 14 after immunization.

### 4.3. In Vitro Cytokine Assay Using EILSA

Cervical draining lymph nodes were collected on day 14 after immunization and pooled within groups. The draining lymph node cells (2 × 10^5^ cells/well) were cultured in 0.2 mL RPMI 1640 (Sigma Aldrich) containing 10 mM HEPES, 0.1 mM nonessential amino acid, 1 mM sodium pyruvate, 100 U/mL penicillin, 100 µg/mL streptomycin (all purchased from Invitrogen Life Technologies, Carlsbad, CA, USA), 10% fetal bovine serum, and IRBP_1-20_ (10 µg/mL). For the cytokine assays, supernatants were collected after 72 h and analyzed for mouse IFN-γ, IL-6, and IL-17 with ELISA kits (Biolegend, San Diego, CA, USA).

### 4.4. Reverse Transcription and Quantitative Polymerase Chain Reaction (PCR)

For quantification of gene expression in the retina, the retina was collected from either eye per mouse. Total RNA from the retina and cervical draining lymph node cells was extracted using Isogen (NIPPON GENE, Tokyo, Japan) and reverse transcribed using High-Capacity RNA-to-cDNA™ Kit (Thermo Fisher Scientific, Waltham, MA, USA). RT reaction had a final volume of 20 μL (5.0 μL total RNA and 15 μL RT reaction solution). The thermal cycling condition consisted of 1 cycle (37 °C for 60 min; 95 °C for 5 min; 4 °C hold). PCR was performed using 2×TaqMan^®^Universal PCR Master Mix (Thermo Fisher Scientific, Waltham, MA, USA) on a QuantStudio 12 K Flex Time PCR System (Thermo Fisher Scientific, Waltham, MA, USA). The reaction condition consisted of 50 °C for 2 min; 95 °C for 10 min; 40 cycles, 2-step cycling (95 °C for 15 s; 60 °C for 1 min) followed by incubation at 4 °C hold. GAPDH was used as an endogeneous control gene for data normalization. If the threshold cycle (Ct) is 40 or more, the value was considered 0 for statistical analysis. Primer and probes were obtained from Thermo Fisher Scientific (Waltham, MA, USA). The assay IDs of each primer and probes were as follow: IFN-γ: Mm01168134_m1, IL-6: Mm00446190_m1, IL-17: Mm00439618_m1, MCP-1: Mm00441242_m1, NQO-1: Mm00500822_g1, HO-1: Mm00516005_m1, and GAPDH:Mm99999915_g1.

### 4.5. Immunohistochemistry

For immunohistochemistry, eyes were enucleated from EAU-induced mice on day 21 after immunization and were fixed with Superfix (Kurabo, Osaka, Japan) and sections were embedded in paraffin (Sigma-Aldrich). Paraffin-embedded sections (10 μm/section) were collected with an autoslide preparation system (Kurabo). Sections were blocked with 5% goat serum in 1× phosphate-buffered saline (PBS) for 1 h at room temperature, and then incubated with primary antibodies in 1XPBS for 24 h at 4 °C. Primary antibodies used were rabbit anti-ionized calcium binding adaptor molecule 1 (Iba-1) (Wako, Osaka, Japan, 1:1000), rabbit anti-GFAP (Dako Cytomation, Santa Clara, CA, USA, 1:200), rabbit anti-mouse IFN-γ (Novus Biologicals, Centennial, CO, USA, 1:500), and rabbit anti-mouse IL-17 (Abcam, Cambridge, UK, 1:500). Sections were then incubated with secondary Abs (Alexa Fluor 488-conjugated anti-rabbit, (Thermo Fisher Scientific, 1:1000) for 1 h at room temperature and counterstained with DAPI (Thermo Fisher Scientific). Images were acquired with a confocal microscope (LSM700, Zeiss, Oberkochen, Germany). We quantified the GFAP positive area or Iba-1 positive area in the retina with Image J. Briefly, GFAP-positive or Iba-1-positive area of each image was segmented by auto-thresholding with Tsai’s “Moments” method to acquire binary images [34] and the relative area (%) of GFAP positive cells or Iba-1 positive cells in a given area of retina was calculated. Similarly, the relative area (%) of IFN-γ positive cells or IL-17 positive cells in a given area of the retina was calculated using binary images. In addition, to measure the relative area of IFN-γ positive cells or IL-17 positive cells in each part of the retina, the retina was separated into three parts as follows: (1) inner-retina (ganglion cell layer~inner plexiform layer), (2) mid-retina (inner nuclear layer~outer plexiform layer), and (3) outer-retina (outer nuclear layer~photoreceptor outer segment). The relative area of IFN-γ positive cells or IL-17 positive cells in each part of the retina was measured and compared between non-immunized WT mice, non-immunized Nrf2 KO mice, immunized WT mice, and immunized Nrf2 KO mice on day 21 after immunization.

### 4.6. Statistical Analyses

Comparison between the two groups was performed using the Student’s *t* test based on the data distribution. ANOVA followed by Bonferroni tests were used for analysis between three or four groups. The significance of the differences was analyzed by SPSS version 28. *p* value of <0.05 was considered to be significant. Data represent mean ± standard deviation.

## Data Availability

Not applicable.
